# Supplementary material for: Predicting visual function by interpreting a neuronal wiring diagram
Source: Nature. 2024 Oct 2;634(8032):113–23. doi: 10.1038/s41586-024-07953-5 (PMC11446822; doi:10.1038/s41586-024-07953-5)
Supplement: Supplementary file 6 — CRF and ERF predictions for individual TmY4 and TmY9 cells. Analogous to Supplementary Data 3, but for TmY target types. Shown are the top four monosynaptic pathways, the strongest pathway passing through each of the top ten intermediary types (ranking from Extended Data Fig. 7), and the trisynaptic pathway Tm1–TmY–Dm3–TmY (see the section entitled Prediction of spatial normalization). [file 41586_2024_7953_MOESM6_ESM.zip › DataS4/TmY9q/720575940622387276.pdf]

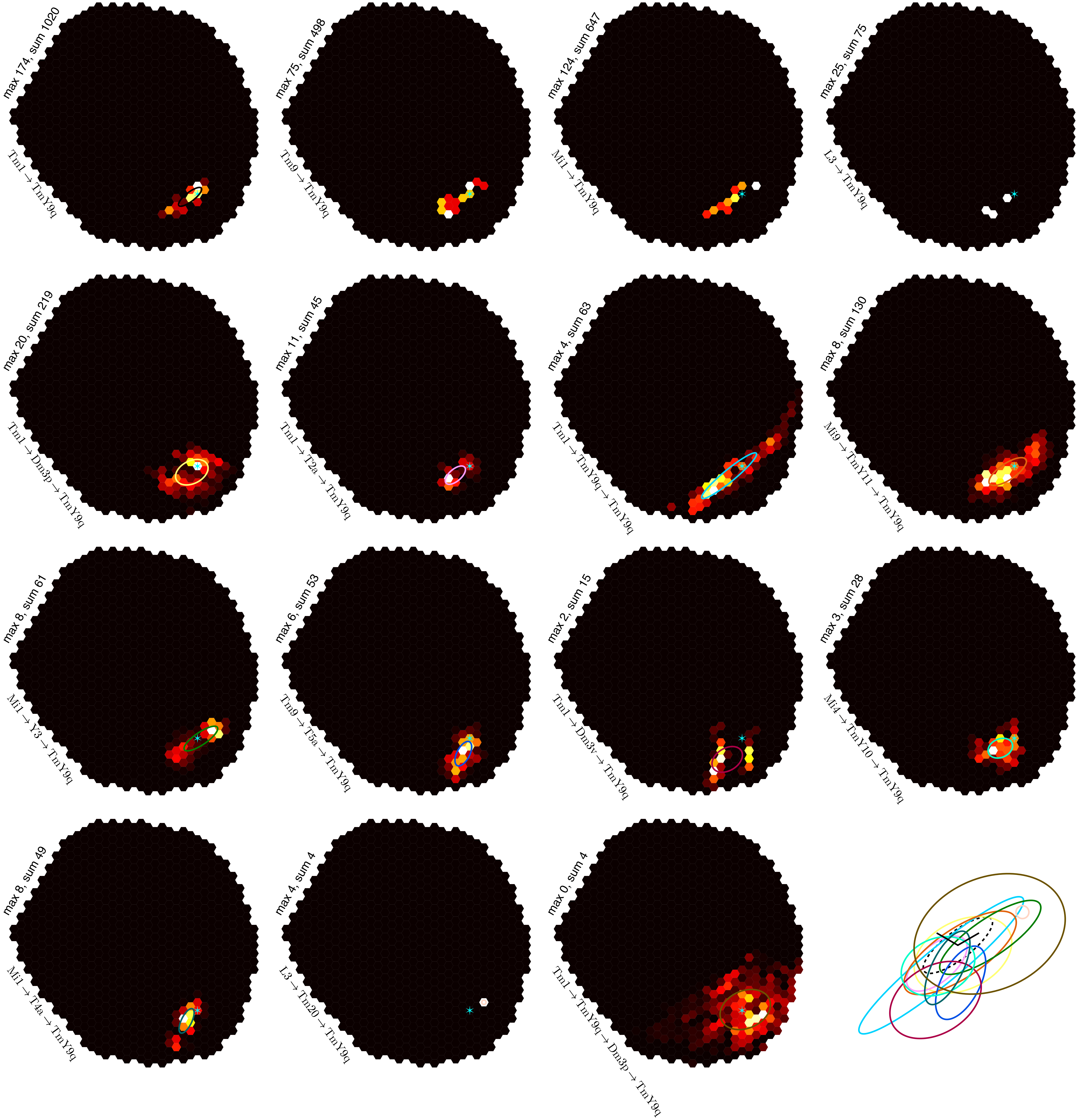
max 174, sum 1020
Tm1 → TmY9q
max 75, sum 498
Tm9 → TmY9q
max 124, sum 647
Mi1 → TmY9q
max 25, sum 75
L3 → TmY9q
max 20, sum 219
Tm1 → Dm3p → TmY9q
max 11, sum 45
Tm1 → T2a → TmY9q
max 4, sum 63
Tm1 → TmY9q → TmY9q
max 8, sum 130
Mi9 → TmY11 → TmY9q
max 8, sum 61
Mi1 → Y3 → TmY9q
max 6, sum 53
Tm9 → T5a → TmY9q
max 2, sum 15
Tm1 → Dm3v → TmY9q
max 3, sum 28
Mi4 → TmY10 → TmY9q
max 8, sum 49
Mi1 → T4a → TmY9q
max 4, sum 4
L3 → Tm20 → TmY9q
max 0, sum 4
Tm1 → TmY9q → Dm3p → TmY9q
